# A pilot study of implementing an adapted model for integration of interventions for people with alcohol use disorders in Tanzanian primary healthcare facilities

**DOI:** 10.1186/s12913-024-10687-9

**Published:** 2024-03-27

**Authors:** Dorothy Mushi, Charlotte Hanlon, Candida Moshiro, Joel M Francis, Merga B. Feyasa, Solomon Teferra

**Affiliations:** 1 Department of Psychiatry and Mental Health, Muhimbili University of Health and Allied Science, United Nations Road, P.O. Box 65001 Dar es Salaam, Tanzania; 2 Department of Psychiatry, School of Medicine, College of Health Sciences, Addis Ababa University, Addis Ababa, Ethiopia; 3 Centre for Innovative Drug Development and Therapeutic Trials for Africa (CDT-Africa), College of Health Science, Addis Ababa University, Addis Ababa, Ethiopia; 4 Centre for Global Mental Health, Health Service and Population Research Department, Institute of Psychiatry, Psychology, and Neuroscience, King’s College London, London, UK; 5 Department of Epidemiology and Biostatistics, Muhimbili University of Health and Allied Sciences, Dar es Salaam, Tanzania; 6 Department of Family Medicine and Primary Care, Faculty of Health Sciences, Witwatersrand University, Johannesburg, South Africa; 7 Division of Epidemiology and Biostatistics, Department of Global Health, College of Medicine and Health Sciences, Cape Town, South Africa; 8 Department of Statistics, College of Natural and Computational Sciences, Addis Ababa University, Addis Ababa, Ethiopia

**Keywords:** Alcohol use disorders, Adapted model, Implementing, Interventions for alcohol use disorders, Integration, Primary health care, Tanzania

## Abstract

**Background:**

Ensuring that evidence-based interventions for people with alcohol use disorders (AUD) are acceptable, effective, and feasible in different socio-cultural and health system contexts is essential. We previously adapted a model of integration of AUD interventions for the Tanzanian primary healthcare system. This pilot study aimed to assess the impact on AUD detection and the acceptability and feasibility of the facility-based components of this model from the perspective of healthcare providers (HCPs).

**Methods:**

This mixed-methods study comprised a pre-post quasi-experimental study and a qualitative study. The integrated model included training HCPs in managing AUD, introducing systematic screening for AUD, documentation of AUD service utilization, and supportive supervision. We collected information on the number of people identified for AUD three months before and after piloting the service model. A non-parametric trend test, a distribution-free cumulative sum test, was used to identify a change in the identification rate of AUD beyond that observed due to secular trends or, by chance, three months before and after implementing the integrated AUD facility-based interventions. The Mann-Kendal test was used to assess the statistical significance of the trend. We conducted three focus group discussions exploring the experience of HCPs and their perspectives on facilitators, barriers, and strategies to overcome them. The focus group discussions were analyzed using thematic analysis.

**Results:**

During the pre-implementation phase of the facility-based interventions of the adapted AUD model, HCPs assessed 322 people for AUD over three months, ranging from a minimum of 99 to a maximum of 122 per month. Of these, 77 were identified as having AUD. Moreover, HCPs screened 2058 people for AUD during implementation; a minimum of 528 to a maximum of 843 people were screened for AUD per month for the three months. Of these, 514 screened positive for AUD (AUDIT ≥ 8). However, this change in screening for AUD was not statistically significant (p-value = 0.06). HCPs reported that knowledge and skills from the training helped them identify and support people they would not usually consider having problematic alcohol use. Perceived barriers to implementation included insufficient health personnel compared to needs and inconvenient health management information systems. HCPs proposed strategies to overcome these factors and recommended multisectoral engagement beyond the health system.

**Conclusions:**

Although the change in the trend in the number of people screened for AUD by HCPs post-implementation was not statistically significant, it is still feasible to implement the facility-based components of the adapted integrated AUD model while addressing the identified bottlenecks and strategies for implementation. Therefore, a large-scale, adequately powered implementation feasibility study is needed. Findings from this study will be used to finalize the adapted model for integrating AUD interventions for future implementation and larger-scale evaluation.

**Supplementary Information:**

The online version contains supplementary material available at 10.1186/s12913-024-10687-9.

## Introduction

The global disease burden attributable to the harmful use of alcohol is substantial [1]. The unmet need for care for people with AUD is also high, particularly in low and middle-income countries (LMICs) [2–3]. For instance, in LMICs, the population treatment gap for AUD is estimated to be between 94.9% and 97.2% [4]. In sub-Saharan Africa (SSA), approximately one in five people attending healthcare facilities meet the criteria for an AUD [5–9]. In Tanzania, nearly one in every four people (23.9%) receiving care in primary health care screened positive for probable AUD, and yet primary health care (PHC) workers detected only one person (0.3%) with AUD [10]. The negative consequences of untreated AUD are considerable, including poor physical and mental health, disability, premature mortality, poor socioeconomic status, and poverty [11–15].

In response to this treatment gap, the World Health Organization (WHO) launched the Mental Health Gap Action Program (mhGAP), which includes an evidence-based intervention guide for the management of priority mental, neurological, and substance use (MNS) conditions in non-specialized healthcare settings [16–17]. A broad range of evidence has accrued to support this model of mhGAP integration in diverse settings [18]. However, the limited focus has been on the impact on people with alcohol use disorders. Adaptation is essential to ensure that these packages of care are effective, sustainable, acceptable, and feasible in different socio-cultural and health system contexts [17, 19–20]. Some countries in sub-Saharan Africa (SSA) have developed and implemented mental health care plans, frameworks, and models that guide the integration of mental health care using mhGAP, including for AUD, in PHC and other settings [20–22]; however, there are no studies from Tanzania. Previous studies from Tanzania have shown that there is a high unmet need at the population level for interventions to address AUD. We previously worked with stakeholders to adapt an integrated AUD care model based on the Tanzanian socio-cultural and health system context [23]. The adapted model comprises integrated community, facility, and organizational levels AUD interventions. In the pilot study presented in this paper, we aimed to assess the impact of the facility-based components of this AUD integration model on AUD detection and to explore acceptability and feasibility from the perspective of healthcare workers.

## Methods

### Study design, setting, participants, and description of the piloting

The methods have been reported in the published study protocol [24]. We used a mixed-methods study comprising a quasi-experimental pre-post-test design and a qualitative study.

### Study setting and participants

The study setting was the Moshi district council, one of the seven administrative districts in the Kilimanjaro region, which is located around 530 km from the main economic city of Dar es Salaam (the former capital city of Tanzania). The Moshi district council has 88 dispensaries, eight health centers (six government-run, two private), and two district hospitals. Dispensaries are the first contact point for basic health services in the community. Tanzania’s health services are provided through the dispensary, health center, district, regional, and tertiary hospitals. The first three levels (dispensary, health center, and district) constitute the Tanzanian PHC system. The study was conducted in all six government-run health centers in the study setting. The study area was selected based on previous studies of AUD in Tanzania, which found that alcohol use (68.0–70.0%) and problematic alcohol use (20.0–47.0%) were highly prevalent in the community [25–26]. Based on these findings, it was recommended that access to interventions for AUD should be expanded [25–28].

### Brief description of the adapted integrated AUD model

The study team consulted with stakeholders and used Theory of Change workshops to develop a model for integrating AUD interventions in the Tanzanian PHC system [23]. The adapted model is a structured framework that includes the evidence-based AUD interventions and their delivery strategy, objective, and expected outcomes at community, facility, and organizational/administrative levels.

The facility-based AUD interventions were piloted in the current study, including capacity building of healthcare providers on AUD management, systematic screening to increase identification of people with AUD, supportive supervision of trained healthcare providers, and documentation of AUD services. (Table 1**)** briefly describes these integrated AUD interventions; more details are available in the protocol [24]. The training content included the causes/contributing factors for AUD, screening, managing, and data recording for AUD [29–32]. Training methods included interactive, participatory techniques and practical sessions.

**Table 1:** Description of the implemented integrated AUD facility-based intervention for the adapted AUD model

Intervention	Description	Contents
Training in management of AUD	The six-day training package was adapted from the WHO guidelines [29–31] and training packages by the program for improving mental health [32] to the Tanzanian context, such Tanzanian case scenarios and examples were used for practical and discussion sessions. Facilitators, psychiatrists, and clinical psychologists.	(i)screening of AUD using a single question and AUDIT, (ii)treatment of AUD (psychoeducation about AUD, motivation interviewing, plan for a follow-up visit, and referral), and (iii) documentation of AUD services.
	A pre and post-test questionnaire	A questionnaire asks about knowledge on causes, risk factors of alcohol use and AUD, and management of people with AUD.
Screening of people with AUD	HCPs, during routine attendance/assessment of the outpatient seeking health care services, also screen for alcohol use	A single question to identify the need for full screening with AUDIT. AUDIT enquires more about frequent alcohol patterns and sub-types of problematic alcohol use, planning AUD treatment accordingly.
Management of people AUD	Based on the AUDIT score, HCPs provide treatment to people identified with AUD as s per the WHO recommended approach for identification and management of AUD.	(i)Psychoeducation about AUD is provided as health education routinely provided before people receive health services. (ii)Brief interventions using motivational interviews, and (iii)a plan for follow-up care was provided to people with harmful and hazardous AUD. (iv)People with dependent AUD and those needing specialized care were referred to the district hospital.
Supportive supervision	The district mental health coordinator leads the study team’s monthly supportive supervision. The goal of supervision was to provide in-service mentorship for AUD screening, management, data collection, and other comorbid mental health conditions.	A routine adapted supervision form was used to assess (i) administrative issues and (ii) clinical skills in diagnosis and treatment plans for AUD.
Documentation of AUD services	A data recording form was adapted in consultation with the HCPs and district mental health coordinator. A form was limited to the general description and diagnosis; An adapted form ensures a standardized and user-friendly approach.	An adapted routine form was used to document the following information (i) a general description, (ii) a number of people who received screening for alcohol use, and (iii) a number of people who received intervention for AUD and information on referral to specialized care.

### Data collection tools and approach

#### Identification of AUD among people attending PHC

As part of routine data collection and monitoring, a facility data coordinator collects data on people with problematic alcohol use, prepares a monthly facility report, and sends it to the district mental health coordinator (MHC). The research team obtained this aggregated information from the monthly reports submitted to the district MHC for the three months before intervention until three months after implementation. These data were routinely collected and submitted to the district MHC, therefore, this reflected usual practice. The routine data collection form contains information on (i) general description, (ii) screening for alcohol use, (iii) interventions for AUD, and (iv) information on referral to specialized care.

#### Implementation of AUD interventions

Healthcare providers received training for the management of AUD that aims to support them in acquiring skills for screening of alcohol use, interventions for AUD and its associated co-morbidities. The training package contains materials from the following manuals: (i)WHO. mhGAP training manuals for mental, neurological, and substance use disorders in non-specialized health settings, version (ii) WHO Manual for Alcohol Use Disorders Identification Test, (iii) WHO Brief Intervention Package for Hazardous and Harmful Drinking, and (iv) Programme for Improving Mental Health Care (PRIME) training packages for HCPs (http://www.prime.uct.ac.za/prime-tools) [29–32], including motivational interviewing techniques adapted for the Tanzanian primary care context. The training was facilitated by the psychiatrist (principal investigator and lecturer) and a clinical psychologist. The training was conducted for five days, using interactive, participatory methods involving role-playing. Healthcare providers then screened for AUD in people presenting to PHC in adult outpatient clinics. They provided treatment according to the AUDIT score using a stepped-care approach (screening for AUD, brief interventions, and referral to specialized care based on the alcohol use disorders identification score) [31]. People scoring 20–40 on the AUDIT, depending on the severity of physical dependence, were referred to the district hospital to access assisted detoxification and other necessary interventions, following the usual referral practice.

HCPs used an adapted routine form to collect the following quantitative data: (i) general description, (ii) screening for alcohol use, (iii) interventions for AUD, and (iv) information on referral to specialized care. A facility data coordinator collected all data collection forms, prepared a monthly facility report, and sent them to the district mental health coordinator, therefore reflecting usual practice.

### Supportive supervision

The study team worked in collaboration with the district supervision team that conducts supervision for mental health services routinely. Approximately two to four HCPs participated in each health center. Three supportive supervision sessions were conducted while implementing the integrated AUD facility-based interventions. During supportive supervision, the discussed and documented administrative issues and clinical skills in diagnosis and treatment plan for AUD were recorded in a routine adapted supervision form, including difficulties in content and flow of screening questions. Moreover, observed challenges were discussed, such as documentation in the adapted routine form and strategies to encounter them.

### Data analysis

Descriptive summaries of the data were obtained. A non-parametric trend test, a distribution-free CUSUM test [33], was used to identify the change point in a series of data, to identify a change in the identification rate of AUD among people attending PHC beyond that observed due to secular trends or by chance, three months before and after implementing the integrated AUD facility-based interventions. The point at which the maximum value of cumulative sum (CUMSUM) occurs will indicate the change point in the data. If the maximum value is equal to or greater than the critical value, it indicates the statistical significance of the change point. Another nonparametric trend test, the Mann-Kendal test, was used to see the statistical significance of the trend. The Mann–Kendall trend test, being a function of the ranks of the observations rather than their actual values, is not affected by the actual distribution of the data and is less sensitive to outliers. A 5% significance level was used as a cut-off for a statistically significant change. The data were managed and analyzed using ‘*trend change’* and ‘*trend’* packages in R software (R, version 4.3.1).

### Qualitative study

We explored (i) the experience of HCPs while piloting the integrated model and (ii) any encountered facilitators, barriers, and strategies used to overcome them. We developed an interview guide for focus group discussions (FGDs) (supplementary file). The participants were selected purposively involved all healthcare providers and heads of the facilities who participated in the piloting of the integrated AUD interventions model. The FGDs were recorded, transcribed in Swahili, and translated into English. Thematic analysis was conducted. The data were managed using qualitative data analysis software (Nvivo, version 12).

### Ethical considerations

The study was reviewed and approved by the institutional review boards (IRB)of Addis Ababa University College of Health Sciences in Ethiopia (meeting 04/2019; protocol number: 023/19/psyc) and the Muhimbili University of Health and Allied Sciences in Tanzania (Ref.No.DA.282/298/01.C/). Participants engaged in the focus group discussions provided written informed consent for participation. The screening of AUD was conducted as part of the routine clinical assessment of clients who turn up at health facilities in need of healthcare services.

## Results

A total of 26 clinicians and nurses, including heads of the facilities providing patient services in the government health centers in the study area, participated in the study.

### HCPs’ identification of AUD

During the pre-implementation phase of the integrated AUD facility-based interventions of the adapted AUD model, HCPs assessed 322 people for AUD over three months, ranging from a minimum of 99 to a maximum of 122 per month. Of these, 77(23.9%) were identified as having AUD (Table 1). Twenty HCPs received training on identifying and managing AUD. During the implementation of the integrated AUD facility-based interventions of the adapted model, HCPs screened 2058 people for AUD. A minimum of 528 to a maximum of 843 people were screened for AUD per month for the three months. Of these, 514(25.0%) screened positive for AUD (AUDIT ≥ 8). They all received brief interventions on the day they were identified, and plans were made for ongoing follow-up. Nine (1.8%) were referred to the district and regional hospital for further specialized care, such as detoxification (Table 2).

**Table 2:** Number of people screened by the healthcare providers Pre and post-implemented integrated AUD facility-based intervention (2022)

**Pre-implementation**	**June**	**July**	**August**	**Total**
Number of people screened by HCPs	101	99	122	322
The number identified as having AUD.				77
**Post-implementation**	**September**	**October**	**November**	**Total**
Number of people screened by HCPs	843	528	687	2058
The number identified as having AUD.				514

**Fig. 1:**
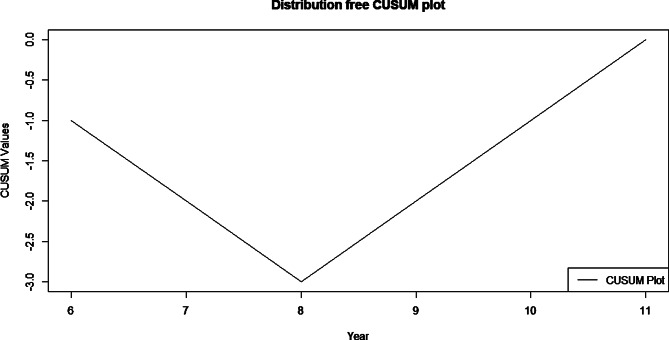
The trend in the number of people screened by the healthcare providers pre- and post-implemented integrated AUD facility-based intervention

Although there was an increasing trend over the months, as per the results from the descriptive analysis, our result from the trend test shows an insignificant (p-value = 0.06) change in trend. Besides, the CUMSUM test also shows that the first month of implementing the intervention (August) is when the change in data points occurred. However, the change was not found to be significant (95% confidence interval CUSUM value 3 is a bit lower than the critical 3.3). See the result in Fig. 1.

### Documentation of AUD services

#### Qualitative results

We conducted three FGDs, two FGDs with the health care providers (*n* = 20) and one with the heads of the six PHC facilities (*n* = 6).

### Feasibility of implementing integrated AUD interventions

In general, HCPs viewed the implementation of the AUD interventions as feasible. The following themes described their experience.

### Training on management of AUD

Most providers commented that knowledge and skills acquired from the screening questionnaires helped them identify and manage people they would not usually have considered having problematic alcohol use, as illustrated by the following quote:

Information we get [healthcare providers] when we screen people with alcohol use disorders guides us on how to help our clients with their problematic alcohol use. But clients told us that the information also helped them realize their problematic alcohol use. [participant number 6-FGDs one]

Other participants spoke of the benefits of training.

For example, the session on the consequences of problematic alcohol use helped me [healthcare provider] understand the association between inappropriate alcohol use and physical conditions and the role of healthcare providers in assisting people attending other health services who had problematic alcohol use. [participant number 2- FGDs two]

### Use of alcohol screening tools

Participants described that the questions were initially challenging and took up much of their time, but they became familiar through practice.

In the beginning, it was a bit hard to use the tools, but as time goes it became easy, and it reached a point where I could ask clients without even reading the. [Participant number 3 -FGDs with the head of facilities]

However, some participants had concerns about the number of questions.

You find many clients needing your services; you have screened a client using a single screening tool and indicated that they need further assessment. There, I started to think, should I ask those questions in a full screening tool while several clients are outside waiting for services? It is challenging to decide if I should ask them or not. [participant number 9- FGDs one]

### Data record and management

Participants reported that the health management information system (HMIS) did not capture information on mental health or substance use conditions. Therefore, it added workload because they needed to record their assessment on documented paper.

You find that you have given services to many clients, but you realize you still need to enter data in a data recording form. While for those conditions in the HMIS, helps you remember and chart them. I suggest the Ministry of Health (MOH) put those common mental health conditions in the HMIS. [participant number 5-FGDs head of facilities]

The unavailability of information system indicators for common mental health conditions, including AUD, in HMIS was described as among the barriers to the routine provision of services. Participants reported that if common mental health conditions were in the HMIS, it would facilitate HCPs’ data recording, like for the physical conditions.

I can say that HMIS doesn’t have data on mental health conditions; therefore, we [healthcare providers] face difficulty providing routine mental health services. If it had information for those common mental health conditions, it would help during assessment and data recording. Otherwise, it becomes difficult as we have to fill in the form and sometimes forget it. [participant number 9 FGDs two]

Furthermore, some patients insisted that because the Ministry of Health is introducing the new electronic HMIS, common mental health conditions, including AUD, should be incorporated into the new electronic system. They emphasized the value of such data for advocacy to improve care.

We all agree that our community is experiencing negative consequences due to the excessive use of alcohol. We must have records if we want evidence to show how big the problem is in our community. If we want to achieve this, the MOH should include these common mental health conditions in the newly enrolled electronic data system, which will help us to improve data collection and recording. [participant number 4 FGDs one]

### The usefulness of supportive supervision

Though they received training for identifying and managing people with alcohol use disorder, most participants described that supportive supervision helped them continue learning skills to deliver competent care to identify and manage people with AUD.

Supportive supervision sessions were helpful in that they helped us continue learning skills for AUD management, even though we got training at the beginning. Therefore, it enabled us to deliver competent care to people with AUD. [participant number 1 FGDs one]

On-going mentorship was reported as the facilitator of the integration of AUD services. HCPs described that ongoing mentorship would help deliver competent services and in-services mentorship to others, hence continuity of care for people with AUD services.

If we don’t continue to receive, mentorship will not be competent enough to offer AUD services. As you realize, we could not do them before due to insufficient knowledge and skills, which we have received now, but we still need ongoing mentorship such that we would also help our co-workers. [participant number 6 FGDs heads of facilities]

### Accessibility to AUD services

Participants perceived that integrating AUD interventions in other healthcare services improved access for people with problematic alcohol use who were not specifically seeking help for AUD.

Because AUD services will be provided within other existing health services, people coming for other health services would also get services for their problematic alcohol. People would agree because they would first receive the services, they needed with AUD services. [participant number 3 -FGDs head of facilities]

#### Insufficient health personnel

Many participants were concerned about being overloaded by the work of routine screening and interventions. Participants described that screening questions helped clients to be aware of drinking patterns. However, due to insufficient staff, work was overloaded due to a number of clients needing health services. Sometimes, clients waiting for services started to complain. Therefore, respondents said this would hinder the routine integration of AUD services. When many people were waiting in the OPD, HCPs reported that they summarized screening questions and planned for the next visit.

Screening and motivation questions were easy to use, and clients understood them, which also helped them self-evaluate their drinking patterns. However, work is overloaded due to a staff shortage compared to the number of people needing health care services. If this continues without action being taken, frankly, it will hinder the integration of these services. [participant number 7 FGDs two]

To overcome work overload, participants suggested staff recruitment while providing AUD services in group sessions instead of individual sessions.

The government had to recruit health staff to intervene in the problem; however, in the meantime, let’s offer AUD services in a group session to minimize work overload. We have experience with other health services provided in a group, for example, the adolescent women’s youth group. [participant number 5 FGDs two]

### Together we can

HCPs perceived that integrating AUD improves access to AUD services by making these services more convenient for patients. However, many people in the community with problematic alcohol use do not come to the facilities. Therefore, we need to work as a team with other sectors to help these people access care.

If will work together with other stakeholders, including local government authority, law enforcement, education, and non-government organizations that support other health services in the community, will help people with problematic alcohol use who don’t come to facilities, particularly youth. [participant number 4 FGDs heads of facilities]

Other participants described further that education about alcohol use and associated consequences in schools and other health education programs and forums would be helpful. Therefore, the Ministry of Education and stakeholders working in a community should also be involved.

We, as HCPs, cannot manage to go to the communities and give education about alcohol use and AUD. We should work with other stakeholders in the communities. [participant number 8 -FGDs two]

Moreover, participants reflected that the law that controls the time to open and operate alcohol shops/places should be properly enforced as a preventive measure.

When we were younger, the law controlling the operation of the alcohol business was implemented. You cannot find people taking or doing alcohol business against the allotted time. But nowadays, people take and sell alcohol very early in the morning and open it, and no one acts. The law enforcement sector should revise the law to suit the current situation and implement it. [participant number 3 FGDs two]

## Discussion

This study used a mixed methods approach to assess the impact of the facility-based interventions of the adapted AUD integration model on AUD detection. It explored acceptability and feasibility from the perspective of healthcare workers.

In comparison to the pre-implementation phase, the number of people screened for AUD by healthcare providers increased during the implementation phase of the integrated AUD facility-based interventions of the adapted AUD model. However, a change in the number of people screened for AUD by HCPs after implementing integrated AUD facility interventions was not statistically significant. In general, HCPs viewed it as possible to implement these integrated facility-based AUD interventions. Also, they identified facilitators, barriers, and strategies to overcome them.

Among other interventions of the adapted AUD model was training on identifying and managing AUD. Healthcare providers reported that it helped them to deliver competent care to people seeking other healthcare services identified to have AUD. This reflected the recommendation by the WHO mhGAP program on integrating evidence-based interventions in non-specialized care to reduce the treatment gap for mental, neurological, and substance (MNS) use disorders, including AUD [16].

Also, a systematic review of strategies to facilitate integrated care for people with AUD and other drug problems revealed that training is among the effective strategies to build the capacity of HCPs to manage AUD and other drug problems [34]. AUD treatment based on the AUDIT screening scale scores was implemented as intended. HCPs offered the following AUD interventions: psychoeducation about AUD, motivational interviewing, a plan for follow-up care, and referral to specialized care. Thus, the WHO recommendation for a stepped-care approach for identification and managing AUD was workable in this setting [30–31]. The stepped care approach for managing people with AUD facilitates person-centered care and access to appropriate services based on needs assessment [35].

Supportive supervision was proposed as an essential component of the AUD intervention of the adapted model. HCPs reported that it helped to discuss administrative and clinical issues and challenges in implementing integrated AUD interventions in facilities. Moreover, they identified it as an essential component that would continue to build HCPs’ to deliver competent care to people with AUD. Similar to other studies in Sub-Saharan Africa [20, 23].

HCPs reported that the current health management information system (HMIS) in Tanzania did not have the features required to capture data for AUD. Therefore, this was perceived to hinder the implementation of the integration of facility-based intervention components of the adapted AUD model. This was also reported in another study in Eastern Africa, which reported the inconvenience of HMIS for data records of mental health conditions, including AUD [20]. During the FGDs, HCPs recommended the ongoing expansions of the Tanzanian HMIS to include features to capture information for AUD services in general healthcare settings.

A shortage of HCPs comparable to the number needed was reported to add workload, hence a barrier in providing AUD screening to people seeking other health services. This finding accords with studies on integrating mental health services in PHC, patients with cancer, and other non-communicable diseases, respectively [36–38].

Healthcare providers recommended multisectoral engagement, such as law enforcement, local government authority, and police, supporting and enhancing the implementation of integrated facility-based AUD intervention. The involvement of law enforcement was recommended to reinforce the laws for controlling and regulating alcohol use. Moreover, the HCP recommended needs for other supportive care, including spiritual, alternative medicine, and traditional healing. Likewise, the same recommendations were reported in other studies on integrating mental health into PHC in South Africa and Uganda [39]. Also, studies in Ethiopia recommended the need to engage other sectors beyond the medical realm to enhance the district mental healthcare plan’s acceptability [19] and study on brief intervention for alcohol use in Ethiopian primary care [40].

### Limitations

Our study has limitations; we only implemented the facility-based integrated AUD intervention of the adapted integrated AUD model. We could not implement the community and organizational aspects of the adapted AUD model. We acknowledge that primary healthcare services are interdependent, interrelated, and interconnecting within the community and system/organizational levels and that greater impact could be achieved by implementing these aspects. The observation time was limited; therefore, we could not conclude whether implementing the integrated facility-based AUD interventions model could increase or decrease the screening of people with AUD, capturing the variation occurring at regular time intervals, such as time of year or day of the week, For instance, in a study area due to the small land. During the farming period for the staple food, many people move to farm places and return to their residences after harvest. We acknowledge that seasonality effects in time series analyses of health data are common [41], for example, due to natural effects and outbreaks. The generalizability of the results could be limited because the study was conducted in public facilities in the Moshi district. Lastly, we did not interview people with AUD to explore their experiences of care.

## Conclusions

Even though a change in the trend in the number of people screened for AUD by HCPs after implementing integrated AUD facility interventions was not statistically significant, it is still feasible to implement the facility-based components of the adapted integrated AUD model while addressing the identified bottlenecks and strategies for implementation. Therefore, there is a call for a large-scale implementing feasibility study. Moreover, these results will be used to finalize the adapted model for integrating AUD interventions for future implementation and larger-scale evaluation.

### Electronic supplementary material

Below is the link to the electronic supplementary material.

Supplementary Material 1

## Data Availability

All data used to write this paper is summarized in tables, figures, or within text in the article. Please get in touch with the corresponding authors should there be a need for this study.
