# Supplementary material for: A pilot study of implementing an adapted model for integration of interventions for people with alcohol use disorders in Tanzanian primary healthcare facilities
Source: BMC Health Serv Res. 2024 Mar 27;24:385. doi: 10.1186/s12913-024-10687-9 (PMC10976770; doi:10.1186/s12913-024-10687-9)
Supplement: Supplementary file 1 — Supplementary Material 1 [file 12913_2024_10687_MOESM1_ESM.docx]

**Focus group discussion interview guide, A pilot study of implementing an adapted model for integration of interventions for people with alcohol use disorders in Tanzanian primary healthcare facilities**

**Introductory Script**: Good morning/afternoon/evening. Thank you for participating in this focus group discussion. Before we begin, I would like to give you some background on the rationale for this research. This study aims to assess the impact of the facility-based interventions of the integrated model for alcohol use disorders (AUD) on alcohol use disorder detection and explore we aimed to assess the impact of the facility-based components of this AUD integration model on AUD detection and to explore acceptability and feasibility from the perspective of healthcare workers.

The discussion will be conducted to explore (1) providers’ experiences in piloting the adapted model of integrated care for people with AUD and (2) the experienced facilitators, barriers, and strategies that were used to overcome them.

Before we begin, I want to remind you that participation in this discussion is voluntary, and anything you say will be kept confidential. With your authorization, responses will be audio recorded, though they will only be shared with other people as part of a summary report, with no names or other identifying information. You can stop the discussion at any point or pass on questions you would prefer not to participate. Before we get started, does anyone have any questions for us?

1. How are you today?

2 . Let's begin by walking through the piloting of an adapted model of integrated care for AUDs in primary healthcare facilities

- What aspects were easy?
- What aspects were difficult?
- Why was that?
- How did the new program fit with your existing daily workflow/plan?
- What could improve this?

3. What has been your experience delivering the integrated care model for AUDs in primary health care facilities?

- Has it caused any impact on your professional

(i)How did you feel when you asked people about their alcohol use?

Were there times when you felt uncomfortable?

Why?

What would have helped?

How acceptable is it to ask people about their alcohol use?

Anybody else?

- Is there anybody who has a different view?

(ii) How did you feel about speaking to people about alcohol problems?

What were the good parts?

What were the bad parts?

Anyone else who has a different experience?

(iii) How supported do you feel in delivering the integrated care model for people with AUDs?

- How about a supervision session?
- How might they be improved?

1. What challenges did you experience during the piloting of the model?

- Was there anything that made it difficult for you to deliver care for people with AUDs?
- How did you overcome these challenges?
- Is there anything that can be done to make it easier for you to deliver care for people with AUDs?

1. What factors facilitated the piloting of the model?

- What could improve them?
- Anyone else?
- Is there anybody who has a different view?

1. What, if any, are your thoughts about your experience in piloting a model of integrated care for AUDs in primary healthcare facilities?
